# A Time Series Study for Effects of PM_10_ on Coronary Heart Disease in Ganzhou, China

**DOI:** 10.3390/ijerph20010086

**Published:** 2022-12-21

**Authors:** Tingting Liu, Hui Huang, Gonghua Hu

**Affiliations:** 1 School of Public Health and Health Management, Gannan Medical University, Ganzhou 341000, China; 2 Key Laboratory of Prevention and Treatment of Cardiovascular and Cerebrovascular Diseases of Ministry of Education, Gannan Medical University, Ganzhou 341000, China

**Keywords:** respirable particulate matter, PM_10_, coronary heart disease, GAM, DLNM

## Abstract

Objective: To investigate the effect of PM_10_ exposure in low concentration areas on the daily hospitalized patients with coronary heart disease. Methods: Daily air quality monitoring data, meteorological monitoring data and daily hospitalization data of coronary heart disease during 2019–2021 in Ganzhou, China were collected. Generalized additive model and distributed lag nonlinear model were used to evaluate the association between environmental PM_10_ and daily hospital visits for coronary heart disease. Stratified by sex and age to see their potential impact on this association. Results: PM_10_ exposure was correlated with an increased risk of hospitalization in coronary heart disease patients. Single-pollutant model analysis shows that at the day of lag1, for every 10 µg/m^3^ increase in PM_10_, the risk of coronary heart disease hospitalization increased by 1.69% (95%CI 0.39~3.00%); Subgroup analysis showed that females and older adults (>65 years) were more sensitive to PM_10_ exposure. In addition, in the dual-pollutant model, by adjusting other pollutants (including SO_2_, CO and O_3_), it was found that the relationship between PM_10_ exposure and coronary heart disease hospitalization was robust. And with changing the model’s degree of freedom was still robust. Conclusion: Short-term exposure to low concentrations of PM_10_ is associated with hospitalization for coronary heart disease. These results are important for local environmental public health policy development, so as to protect vulnerable populations.

## 1. Introduction

According to the World Health Organization (WHO), more than 99% of people breathe air that exceeds WHO air quality limits, and exposure to air pollution causes 7 million premature deaths each year, which can lead to lower incomes and productivity. Meanwhile, air pollution is a major environmental problem in developing countries, and particulate pollution is most severe [1,2,3]. Previous studies have shown that exposure to particulate matter can cause certain harm to the respiratory and circulatory systems [4,5,6,7], but most studies have focused on areas with high pollution concentrations. Although there is also literature showing that even if particulate pollution levels do not exceed currently regulated levels, there is still an impact on cardiovascular disease [8], such as the ESCAPE (European Study of Cohorts for Air Pollution Effects) study found a 12% (95%CI 1~25%) increased risk of coronary events for every 10 µg/m^3^ increase in PM_10_ [9].

Coronary heart disease (CHD) refers to plaques formed by the accumulation of some atheroma-like substances in the arterial intima. These plaques gradually increase which can result in arterial lumen stenosis, block blood flow, and cardiac ischemia or hypoxia [10]. CHD is not only affected by factors such as heredity and dietary habits, but also by environmental factors (air pollution, etc.). Several current clinical studies have shown that inhaled air pollutants, especially respirable particulate matter, can enhance inflammation and oxidative stress, thereby causing endothelial dysfunction, favoring the formation of fragile plaques with localized inflammatory infiltration and higher lipid burden, which provides a plausible biological explanation for the association between PM_10_ exposure and CHD [11,12]. Due to the differences in pollutant components in various regions, the extrapolation of the research resulting in various regions is poor. Despite the fact that the PM_10_ concentration in Ganzhou does not exceed the secondary standard of ambient air quality in China (150 µg/m^3^ for average daily concentration) [13], by evaluating the relationship between PM_10_ exposure in ambient air and daily hospitalization rate of CHD in Ganzhou, it will be helpful to understand the impact of low concentrations of PM_10_ on CHD.

Ganzhou is located in the south gate of Jiangxi province, accounting for 23.6% of the province’s area. It has a typical subtropical monsoon climate, with prevailing winter and summer winds, concentrated precipitation in spring and summer, a mild climate, abundant heat, and abundant rainfall, it is one of the key non-ferrous metal bases in China [14]. The local PM_10_ pollution is mainly generated by the combustion of fuel and production and construction in different departments, including transportation, household, and industrial and agricultural production. In addition, the results of the seventh population census show that Ganzhou has a permanent population of about 8.97 million, and the elderly aged over 60 account for about 15%, including about 2.9 million urban population and about 6 million rural population [14]. There is a large gap between urban and rural areas, and the level of urbanization is low, and its ecological environment, economy and society, humanities, science, and education have a certain gap with the developed areas. Due to its unique geographical location, climate environment, and economic and cultural level, all these will affect the occurrence and development of the disease, and there are certain differences with other regions (Supplementary Figure S1).

In this study, the generalized additive model (GAM) and distributed nonlinear lag model (DLNM) were used to quantitatively evaluate the relationship between PM_10_ exposure and the number of daily hospitalized patients with CHD in Ganzhou. Subgroup analysis of gender and age was also carried out to determine the most vulnerable subgroup, so as to provide a scientific basis for further formulating effective environmental regulations and protecting vulnerable populations.

## 2. Methods

### 2.1. Data Collection

The data of daily hospitalization for CHD on 1 January 2019 and 31 December 2021 were obtained from two general tertiary hospitals in Ganzhou: The First Affiliated Hospital of Gannan Medical University and Ganzhou People’s Hospital. These hospitals are the largest in Ganzhou, with more than 2000 people hospitalized due to CHD every year. The patient data collected in the medical record system included age, sex, date of admission, place of residence, and principal diagnosis on discharge coded with the 10th revision of the international classification of diseases (ICD-10: I20–I25). A total of 33,653 CHD inpatients were collected during the study period. Daily air pollution monitoring data during the same period came from Ganzhou Environmental Protection Bureau, mainly collecting data from five state-controlled monitoring points in Ganzhou. The main atmospheric pollutants include the average daily concentration of PM_10_, PM_2.5_, SO_2_, NO_2_, and CO and the maximum 8-h average concentration of O_3_. The average daily temperature and humidity data during the study period were obtained from Ganzhou Meteorological Bureau. There were no missing values for daily air pollutant concentration and meteorological data.

### 2.2. Statistical Analysis

SPSS 26.0 software (IBM corporation, Armonk, NY, USA) was used to organize and analyze the data. The measurement data such as air pollution, meteorological data and daily hospitalization data were first tested for normality: if the normal distribution was satisfied, it was represented by a mean and standard deviation (SD). Otherwise, the minimum value, maximum value, and percentile (P_25_, M, P_75_) are used for statistical description. Spearman rank correlation analysis was used to analyze the correlation between PM_10_, other air pollutants (PM_2.5_, NO_2_, SO_2_, CO, and O_3_), and meteorological factors (temperature and relative humidity). When the correlation coefficient of the two factors |r| > 0.6, to avoid multicollinearity in the subsequent modeling, the two factors should not be included in the model at the same time [15,16].

For the general population, the CHD daily hospitalization data is a small probability event, and its actual distribution approximates the Poisson distribution. Therefore, this study employed GAM and DLNM to fit the relationship between citywide daily PM_10_ concentrations and CHD hospitalization rates. We used natural spline functions to control for confounding factors such as the effects of long-term trend of time, relative humidity, temperature, and weekend effects. Referring to previous studies, the degrees of freedom were set as 3–8 for continuous fitting [17,18]. At the same time, according to Akaike’s information criterion (AIC), the minimum AIC value was the optimal degree of freedom [19,20]. We finally used eight (*df*) as a stable function of time every year to control the fluctuation of hospitalization on the long-term trend, four (*df*) for the average temperature on the day, and three (*df*) indicated the relative humidity. We first used GAM to assess the health effects of PM_10_ at different lag days (single-day lag and sliding-average lag), which generally takes the following form:

(1) $$Log[E(Y_{i})]=\alpha +\beta_{i}X_{i}+ns(T_{mean},df=4)+ns(time,df=8/\mathrm{per}\;\mathrm{year})+ns(RH,df=3)+DOW$$

where: *Y_i_* is the number of CHD inpatients on the day *i*; *E(Y_i_)* is the expected number of CHD inpatients on day *i*; *α* is the intercept; *βi* is the regression coefficient; *X_i_* is the PM_10_ concentration on day *i* (µg/m^3^); ns is the natural spline smoothing function; *T_mean_* is the average temperature of day *i*; time is the date variable; *RH* is the average relative humidity of day *i*; and *DOW* means the day of the week effect, is a phenomenon that leads to periodic fluctuations in the number of hospital visits due to weekends.

The DLNM was used to reflect the exposure–response relationship between PM_10_ concentration and the relative risk of hospitalized patients with CHD. Its basic model is as follows:

(2) $$Log[E(Y_{t})]=\alpha +\beta_{i}Z_{t}+ns(T_{mean},df=4)+ns(time,df=8/\mathrm{per}\;\mathrm{year})+ns(RH,df=3)+DOW$$

where: *E(Y_t_)* means the expected number of CHD inpatient visits at lag day *t* and *Z_t_* is the cross-basis function of PM_10_.

Due to the lag effect of PM_10_ on population health, this study firstly analyzed the influence of PM_10_ on the single-lag day effect (lag0–lag7) and multi-lag day effect (lag01–lag07) of CHD hospitalized patients. Secondly, subgroup analysis was used to explore the relationship between PM_10_ and the number of CHD inpatients in different gender and age subgroups. Thirdly, the exposure-response relationship between PM_10_ and CHD inpatients was described. Finally, the dual pollutant model was further used to analyze the influence of PM_10_ on hospitalization of CHD under the combined action of other factors, so as to evaluate the stability of the model [21]. Meanwhile, the stability of the model was evaluated by changing the degree of freedom of the model.

All statistical analyses were conducted in R 3.6.3 (R Foundation for Statistical Computing, Vienna, Austria) using the “mgcv” and “dlnm” packages. The results were expressed in terms of the percentage increases (excess risk (%)) in CHD hospital admissions for 10 µg/m^3^ increment of PM_10_ and their respective 95%CI. The results of the exposure–response relationship were expressed as the relative risk (RR) of CHD inpatient visits caused by PM_10_ exposure. In this study, two-sided *p* < 0.05 was considered statistically significant.

## 3. Results

### 3.1. Descriptive Statistics

Table 1 presents the descriptive results of ambient air pollutant concentrations, meteorological factors, and daily CHD inpatients in Ganzhou. Between 1 January 2019 and 31 December 2021, a total of 33,653 hospitalizations with CHD were enrolled in the study population. The average daily number of hospitalized patients was 31, including 11,832 females (35.16%) and 21,821 males (64.84%). Older people (age ≥ 65 years) accounted for 56.82% of all the cases. During the study period of 1095 days, the average daily concentration (standard deviation) of air pollutants PM_10_ was 46.42 (22.91) µg/m^3^, PM_2.5_ was 25.97 (13.23) µg/m^3^, SO_2_ was 11.02 (4.99) µg/m^3^, NO_2_ was 19.53 (11.63) µg/m^3^, CO was 1.07 (0.24) mg/m^3^, and O_3_ was 88.19 (37.53) µg/m^3^, all of which did not exceed the daily average concentration limit of the secondary standard of GB3095-2012 “Ambient Air Quality Standard” [13]. Moreover, the daily mean relative humidity and temperature were 72.54% and 21.34 °C, respectively. The daily trend of air pollution, meteorological factors, and the number of CHD inpatients in Ganzhou during the study period are shown in Supplementary Figure S2. It can be seen that the number of CHD patients hospitalized is on the rise year by year in general, and there is a certain volatility. At the beginning of 2020 there is a fluctuation, which may be related to the outbreak of COVID-19. Other factors are seasonal and cyclical fluctuations.

### 3.2. Correlation Analysis

The correlation between meteorological factors and atmospheric pollutants is shown in Supplementary Table S1. Daily concentrations of PM_10_ were positively correlated with all other air pollutants (PM_2.5_, SO_2_, NO_2_, CO, and O_3_, r = 0.18~0.95), negatively correlated with temperature (r = −0.12), and relative humidity (r = −0.45). Except for CO, which was positively correlated with relative humidity (r = 0.27), other factors were negatively correlated with relative humidity. SO_2_ and O_3_ are positively correlated with temperature, but other factors were negatively correlated. Since PM_10_ is strongly correlated with PM_2.5_ and NO_2_ (|r| > 0.6), we did not include it in the model analysis at the same time.

### 3.3. Model Fitting Results

On the basis of controlling long-term trends in time, the “day of the week effect”, temperature, and humidity, PM_10_ was put into the single pollutant model, and single-lag day effect and multi-lag day effect were taken into account. We found that PM_10_ was significantly associated with the risk of CHD hospitalization on the day of lag0 and on the day of lag1. The maximum effect was achieved at the day of lag1, and the risk of CHD hospitalization increased by 1.69% (95%CI 0.39~3.00%) for each 10 µg/m^3^ increase in PM_10_. The multi-lag day effect of PM_10_ was also found to be significantly different in the day of lag0–1, the day of lag0–2, and the day of lag0–3, with a maximum effect of 2.10% (95%CI 0.59~3.62%) at the day of lag0–1, and other statistical results are shown in Table 2. Supplementary Figure S3 indicates that with the extension of lag time, the cumulative lag effect gradually weakens and tends to have no statistical significance. The stratified analysis by gender and age in Table 3 shows that the effect of PM_10_ exposure on female patients is slightly greater than that of male patients, with the estimated cumulative effects (lag0–2) of 2.56% (95%CI 0.61~4.54%) and 1.97% (95%CI 0.22~3.74%) increases in the risk of CHD hospitalization per 10 µg/m^3^ increment of PM_10_, respectively. For younger persons (<65 years), the daily CHD inpatient visits were significantly associated with PM_10_ concentration on the day of lag0. However, for the elderly (≥65 years), the daily CHD inpatient visits were significantly associated with PM_10_ concentration at the day of lag1. Furthermore, in the cumulative lag effect (lag0–2), the risk of hospitalization for PM_10_ exposure was higher in elder persons than in younger persons.

**Figure 1:**
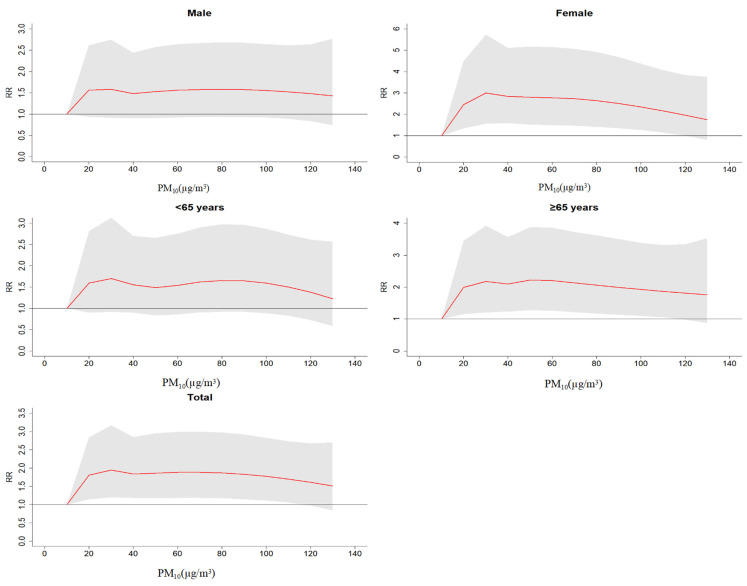
Relative risk (RR) of CHD hospitalization caused by different PM_10_ concentrations.

Figure 1 shows the exposure–response relationship between PM_10_ concentration change and the relative risk of hospitalization for CHD using DLNM and setting the maximum lag day (lag7). The results show four conclusions. (1) The results of the exposure–response relationship is a “nonlinear relation”, and there was statistical significance in females, the elderly (≥65 years), and the total population. (2) When the concentration of PM_10_ was 10–30 µg/m^3^, the risk of CHD hospitalization increased significantly, (3) When the concentration of PM_10_ was 30–100 µg/m^3^, the risk of CHD hospitalization remained at a relatively stable level. (4) When the concentration of PM_10_ > 100 µg/m^3^, the risk of CHD hospitalization slightly reduced.

Table 4 analyzes PM_10_ and SO_2_, CO, and O_3_ for dual-pollutant model. We used the data with the largest single-lag day effect and introduced the above three pollutants at the day of lag1. The results showed that after adjusting for SO_2_, CO, and O_3_ the effect of PM_10_ on the risk of CHD hospitalization was slightly increased by a statistically significant amount, and the percentages of increased risk of CHD hospitalization were 1.95%, 1.78%, and 2.23%, respectively. When adjusting for other pollutants, PM_10_ exposure still increases the risk of hospitalization for CHD, which also reflects the relative stability of our model. In addition, the single pollutant model PM_10_ (lag1) had the strongest effect on daily CHD hospitalization, its sensitivity analyses were mainly evaluated by changing the model degree of freedom (*df*) values (3–8, respectively). As shown in Supplementary Table S2, at the day of lag1, by changing the *df* of the model time and meteorological factors, the ER value of PM_10_ on the daily hospitalization of CHD fluctuated from 1.68% to 2.74%, and the differences were statistically significant (*p* < 0.05). Therefore, the reliability of the model can be judged.

## 4. Discussion

To our knowledge, this is the first study focusing on the short-term effects of PM_10_ exposure on CHD hospital visits in Ganzhou, China. In this study, hospitalization records in Ganzhou from 2019 to 2021 were collected to investigate the relationship between PM_10_ and CHD. We found that exposure to low PM_10_ concentration is an important environmental factor which affects CHD hospitalization. Elevated PM_10_ concentrations were significantly associated with increased CHD hospital visits, especially in females and the elderly (≥65 years). The exposure-response curve of PM_10_ concentration and the relative risk of hospital visits for CHD inpatients showed an almost “nonlinear relation”. Moreover, after adjusting other pollutants and model degrees of freedom, this correlation is still relatively stable.

This study shows that in the single pollutant model, the single-day lag effect of PM_10_ reaches the peak at lag1, and the cumulative-lag effect reaches the peak at lag0–1. At this point, each increase of 10 µg/m^3^ in PM_10_ was associated with an increase of 1.69% (95%CI 0.39~3.00%) and 2.10% (95%CI 0.59~3.62%) in daily CHD hospitalizations, respectively, and with the extension of lag time, the lag effect gradually decreases and tends to have no statistical significance. This result is consistent with previous research results, indicating that exposure to PM_10_ concentration is correlated with the incidence of CHD [22,23]. A study from Jinan, China, showed that the strongest associations between PM_10_ and the risk of myocardial infarction hospitalization occurred at lag0, with an increase of 4.7% (95%CI 4.1~5.2%) [24]. Similarly, PM_10_ exposure increases the risk of hospitalization with CHD, which was observed at lag 0–5 in Hong Kong [RR 1.008 (95%CI 1.005~1.011)] [25]. A quantitative review also showed that the short-term (time lag: 0–5 days) effects of PM_10_ were seen on cardiovascular diseases hospital admission and the percentage changes in the cardiovascular disease admission associated with per 10 µg/m^3^ increase in PM_10_ ranged from 0.5% to 4.8% [26]. Although the effects of PM_10_ on different cities were different, all of them indicated that PM_10_ exposure was associated with hospitalization for CHD. This difference may be due to population susceptibility, climate patterns, topography, long-term air pollution levels and composition, and different study periods in different cities.

The results of the stratified analysis showed that age ≥ 65 years was more sensitive to PM_10_ exposure, which is consistent with previous studies [27]. This may be because the elderly tend to have atherosclerotic plaques and other cardiovascular diseases and thus are exposed to PM_10_ and are more likely to induce their hospitalization. Short-term exposure to PM_10_ is an important trigger for CHD, possibly because of its ability to increase plaque vulnerability, and induce vasospasmodic constriction, platelet activation, and coagulation [28]. Our study finds that females are more sensitive to PM_10_ exposure, and this is consistent with the results of a study in Shenyang [29]. It may be that a relatively smaller physique, greater airway reactivity, and more PM deposition in the lungs may make females more vulnerable to air pollution than males [30]. However, a study in Shanghai, China, found that males were more sensitive to PM_10_ exposure [23]. It is also possible that gender differences between different studies are caused by factors such as study design, sample size, and modeling strategy. Therefore, more evidence is needed to clarify the results.

By understanding the PM_10_ exposure response curve, we found that PM_10_ was positively correlated with daily CHD hospitalizations at low exposure levels, and the slope is steeper when PM_10_ level < 30 µg/m^3^. However, CHD hospitalizations did not increase proportionally with pollutant concentrations. This may be due to the fact that most frail people are already ill and hospitalized before the concentration threshold is reached, so exposure to low PM_10_ concentrations is clearly positively associated with CHD hospitalization. Moreover, people exposed to higher levels of air pollution for a long time may be tolerant of air pollution, which may also weaken the relationship between high PM_10_ concentrations and CHD hospitalizations [31]. In this study, a dual-pollutant model was used to consider the relationship between PM_10_ and CHD hospitalization under the combined effects of other air pollutants. We found that PM_10_ had a statistically significant effect on CHD hospitalization after adjusting for other pollutants, this indicates that the fitting results of our model are relatively stable. At the same time, we further found that our model results are stable by varying the model degrees of freedom.

However, several limitations should be noted in this study. First, as an ecological time-series study, it cannot adjust for individual confounding factors, such as dietary habits, individual economic level, etc. Second, we used the average value of five outdoor monitoring points in Ganzhou to replace the individual exposure level, in addition, during the particular time period we selected (COVID-19), people were more likely to wear masks, which may reduce the inhalation of air pollutants, all of which to some extent underestimated the impact of PM_10_ exposure on CHD hospitalization. Third, it was a single city study, and the samples we collected were small, we did not analyze the relationship between the five types of CHD and PM_10_ one by one. These cases could not replace the incidence of all cases, which had a certain selection bias. Finally, we could not distinguish whether the data collected were from newly admitted patients or not.

In Ganzhou, a subtropical city in southern China, lower PM_10_ concentrations were observed to be associated with daily CHD hospitalizations, although the average daily PM_10_ concentration was below the minimum allowable exposure concentration set by China. This suggests that current standards are insufficient to protect people, especially those with CHD, from the health risks associated with PM_10_ exposure. Furthermore, since PM_10_ composition varies in different regions, we call for a multi-city study on the relationship between PM_10_ and hospitalization for CHD. In the future, it is necessary to further analyze the influence of the composition of fine particulate matter in Ganzhou on CHD hospitalization.

## 5. Conclusions

In summary, this study reveals that elevated PM_10_ is associated with an increase in CHD admissions in the low PM_10_ concentration state and that women and the elderly are more sensitive. In the case of low-level PM_10_ exposure, it is particularly necessary to formulate air pollution prevention and control measures suitable for susceptible groups. In addition, this result helps to further understand the harmful mechanism of air pollution on CHD, and can provide a reference for local environmental governance, which has important public health significance. We can reduce the health hazards of PM_10_ exposure by appropriately reducing the frequency of going out during periods of harsh air, air purification, wearing masks, or lifestyle changes.

## Figures and Tables

**Table 1:** Summary statistics of daily hospitalized patients for CHD, air pollution concentrations and weather conditions in Ganzhou 2019–2021.

Variables	Mean ± SD	Min	P_25_	P_50_	P_75_	Max
Daily hospitalized patients						
Total patients	30.71 ± 14.13	3.00	20.00	29.00	39.00	115.00
Male patients	19.91 ± 9.78	1.00	13.00	19.00	26.00	74.00
Female patients	10.80 ± 5.73	0.00	6.00	10.00	14.00	41.00
Age < 65	13.26 ± 7.06	0.00	8.00	12.00	17.00	43.00
Age ≥ 65	17.45±8.77	1.00	11.00	16.00	23.00	72.00
Pollution concentration						
PM_10_ (μg/m^3^)	46.42 ± 22.91	10.00	29.00	41.00	59.00	137.00
PM_2.5_ (μg/m^3^)	25.97 ± 13.23	4.00	16.00	24.00	34.00	97.00
SO_2_ (μg/m^3^)	11.02 ± 4.99	2.00	8.00	10.00	14.00	30.00
NO_2_ (μg/m^3^)	19.53 ± 11.63	4.00	12.00	16.00	24.00	84.00
CO (mg/m^3^)	1.07 ± 0.24	0.60	0.90	1.00	1.20	2.20
O_3_ (μg/m^3^)	88.19 ± 37.53	4.00	62.00	85.00	111.00	224.00
Meteorological factors						
Temperature (°C)	21.34 ± 8.01	1.50	15.00	22.00	29.00	33.50
Relative humidity (%)	72.54 ± 12.20	33.00	63.00	72.00	82.00	96.00

SD: standard deviation.

**Table 2:** Effects of different lag days on CHD hospitalization risk in a single pollutant model (ER% (95%CI) for 10 µg/m^3^ increment of PM_10_).

Lag Type	Lag Day	ER	95%CI
Single-lag days	lag0	**1.60**	**(0.22~3.01)**
	lag1	**1.69**	**(0.39~3.00)**
	lag2	0.85	(−0.38~2.1)
	lag3	0.05	(−1.14~1.27)
	lag4	−0.08	(−1.26~1.12)
	lag5	0.55	(−0.64~1.75)
	lag6	0.03	(−1.15~1.23)
	lag7	−0.91	(−2.08~0.28)
Multi-lag days	lag0–1	**2.10**	**(0.59~3.62)**
	lag0–2	**2.05**	**(0.46~3.68)**
	lag0–3	**1.72**	**(0.05~3.42)**
	lag0–4	1.47	(−0.27~3.23)
	lag0–5	1.57	(−0.23~3.42)
	lag0–6	1.42	(−0.46~3.34)
	lag0–7	1.14	(−0.82~3.14)

ER means excess risk (%); Bold font indicates statistical significance (*p* < 0.05).

**Table 3:** Effects of PM_10_ on CHD hospitalization risk by gender and age groups, 2019–2021 (ER% (95%CI) for 10 µg/m^3^ increment of PM_10_).

Lag Type	Lag Days	Male	Female	<65	≥65
Single-lag days	lag0	**1.72 (0.21~3.26)**	1.66 (−0.04~3.39)	**2.10 (0.40~3.83)**	1.39 (−0.14~2.95)
	lag1	**1.57 (0.16~3.01)**	**2.14 (0.56~3.75)**	1.54 (−0.05~3.16)	1.93 (0.49~3.38)
	lag2	0.71 (−0.64~2.08)	1.32 (−0.20~2.86)	0.58 (−0.95~2.13)	1.16 (−0.21~2.55)
	lag3	−0.05 (−1.36~1.27)	0.44 (−1.04~1.94)	−0.38 (−1.86~1.13)	0.48 (−0.85~1.83)
	lag4	−0.39 (−1.68~0.92)	0.67 (−0.79~2.16)	−0.73 (−2.19~0.76)	0.51 (−0.81~1.84)
	lag5	0.44 (−0.85~1.75)	0.90 (−0.56~2.39)	0.42 (−1.05~1.91)	0.73 (−0.58~2.06)
	lag6	0.37 (−0.92~1.68)	−0.46 (−1.91~1.02)	0.10 (−1.37~1.59)	0.07 (−1.24~1.40)
	lag7	−0.48 (−1.77~0.82)	−1.57 (−3.01~−0.10)	−1.03 (−2.49~0.45)	−0.73 (−2.03~0.59)
Multi-lag days	lag0–2	**1.97 (0.22~3.74)**	**2.56 (0.61~4.54)**	**2.04 (0.08~4.04)**	**2.25 (0.49~4.05)**
	lag0–5	1.32 (−0.65~3.33)	**2.43 (0.23~4.69)**	1.07 (−1.15~3.35)	**2.15 (0.16~4.19)**
	lag0–7	1.17 (−0.97~3.35)	1.51 (−0.87~3.96)	0.64 (−1.77~3.12)	1.74 (−0.42~3.95)

Bold font indicates statistical significance (*p* < 0.05).

**Table 4:** ER change (95%CI) of CHD hospitalization associated with 10 µg/m^3^ increase of PM_10_ under multiple pollutant models.

Lag Days	Model Type	ER	95%CI
lag1	PM_10_	**1.69**	**(0.39~3.00)**
	PM_10_ + SO_2_	**1.95**	**(0.43~3.49)**
	PM_10_ + CO	**1.78**	**(0.45~3.13)**
	PM_10_ + O_3_	**2.23**	**(0.72~3.76)**

Bold font indicates statistical significance (*p* < 0.05).

## Data Availability

Data were obtained from the electronic medical record system of the First Affiliated Hospital of Gannan Medical College and Ganzhou People’s Hospital, and were available from the hospital records room with the permission of these two hospitals. But the medical system needs to keep patient information confidential, so raw data cannot be made public.

## Supplementary Materials

The following supporting information can be downloaded at: https://www.mdpi.com/article/10.3390/ijerph20010086/s1, Figure S1: Distribution of meteorological monitoring stations and the hospital; Figure S2: Time series graph to show the daily variation of CHD inpatients, concentrations of air pollutants and meteorological factors; Figure S3: Sensitivity analyses showed the effect of PM_10_ on single-day lag and cumulative lag effects on CHD hospitalizations from day0 to day14; Table S1: Spearman’s correlation between air pollutants and meteorological factors in Ganzhou during 2019–2021; Table S2: Effects of PM_10_ on CHD hospitalization at different time, temperature and relative humidity *df* values.
