# Supplementary material for: A Time Series Study for Effects of PM10 on Coronary Heart Disease in Ganzhou, China
Source: Int J Environ Res Public Health. 2022 Dec 21;20(1):86. doi: 10.3390/ijerph20010086 (PMC9819568; doi:10.3390/ijerph20010086)
Supplement: Supplementary file 1 [file ijerph-20-00086-s001.zip › ijerph-1980023-supplementary.pdf]

Supplementary Materials

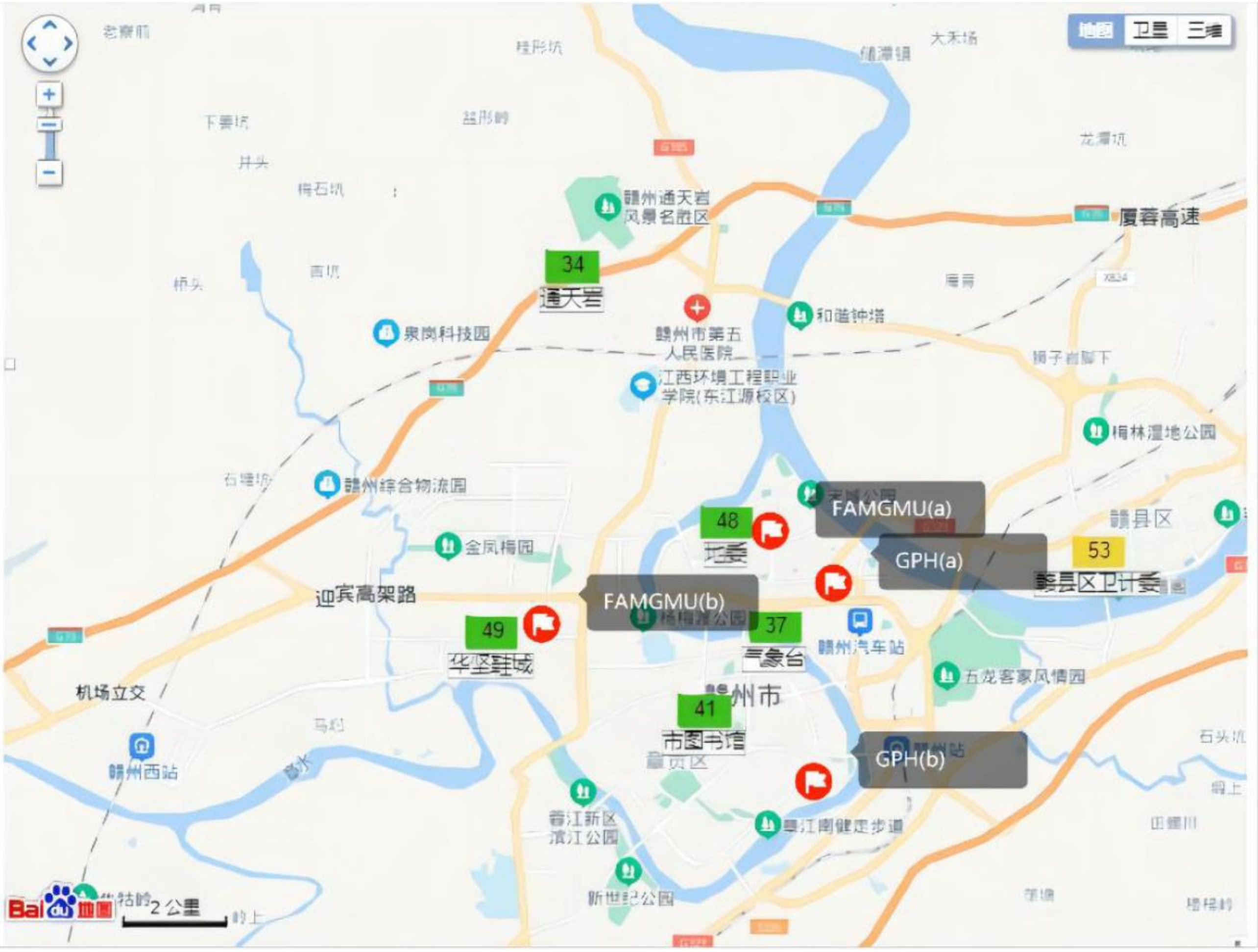

Figure S1. Distribution of meteorological monitoring stations and the hospital.

Notes: The green marks represent monitoring sites and the red flags represent hospitals; FAHGMU: The First Affiliated Hospital of Gannan Medical University; GPH: Ganzhou People's Hospital. a/b: Different hospital districts of the two hospitals.

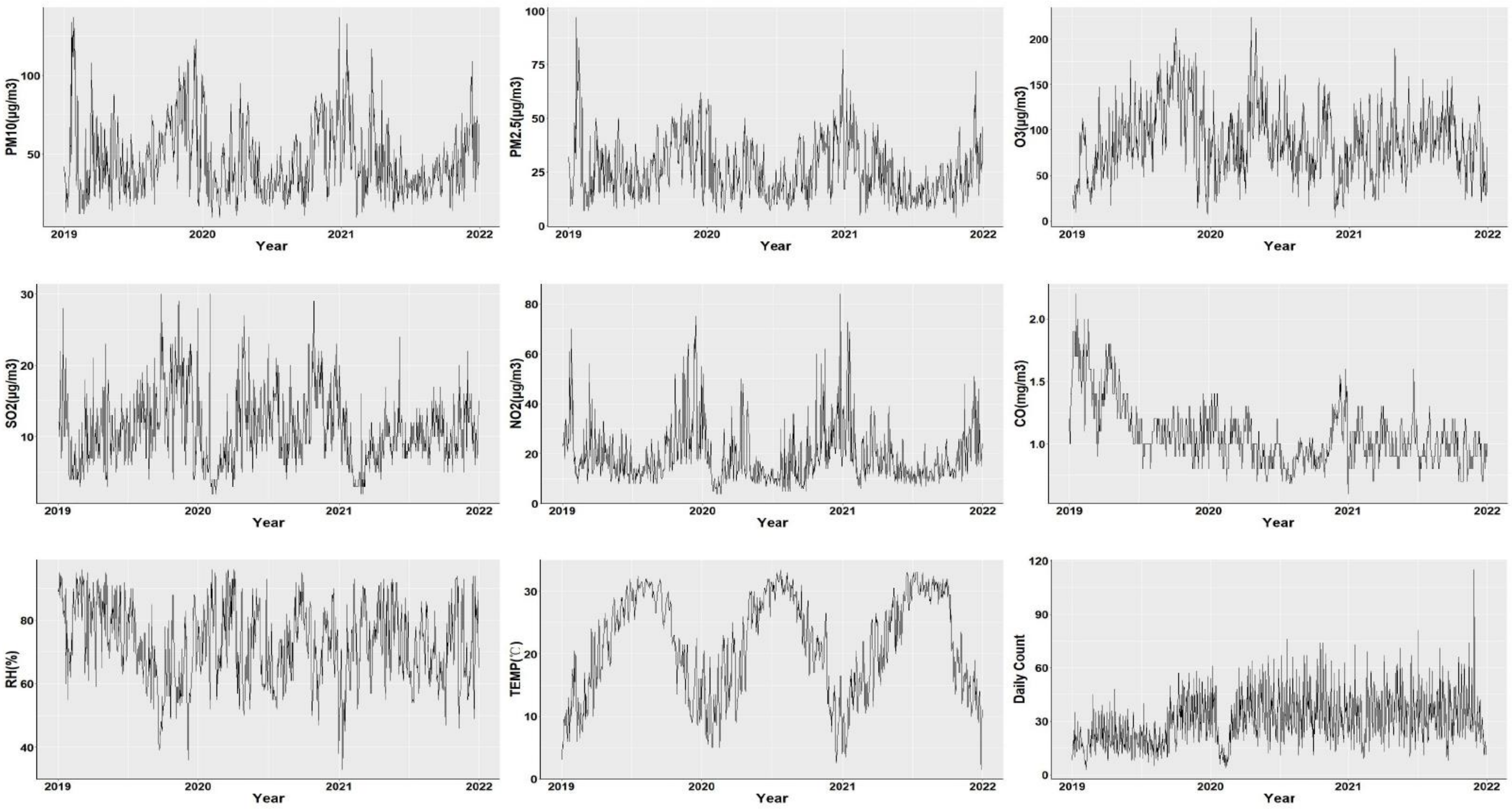

Figure S2. Time series graph to show the daily variation of CHD inpatients, concentrations of air pollutants and meteorological factors.

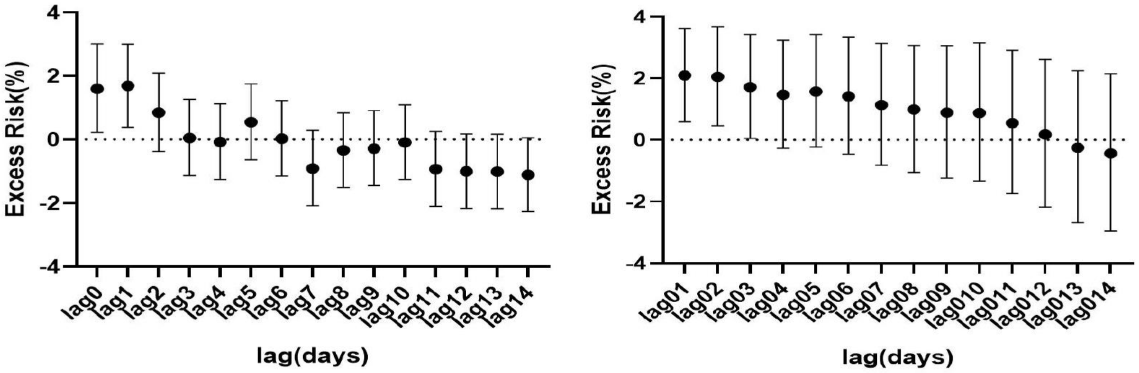

Figure S3. Sensitivity analyses showed the effect of PM_{10} on single-day lag and cumulative lag effects on CHD hospitalizations from day0 to day14.

Table S1. Spearman's correlation between air pollutants and meteorological factors in Ganzhou during 2019–2021.

Factors	PM_{10}	$PM_{2.5}$	SO_2	NO_2	CO	O_3	Temperature	Relative humidity
PM_{10}	1.00	0.95 *	0.55 *	0.67 *	0.18 *	0.50 *	−0.12 *	−0.45 *
$PM_{2.5}$		1.00	0.47 *	0.62 *	0.26 *	0.43 *	−0.20 *	−0.31 *
SO_2			1.00	0.40 *	−0.03	0.44 *	0.23 *	−0.48 *
NO_2				1.00	0.28 *	0.10 *	−0.38 *	−0.14 *
CO					1.00	−0.04	−0.23 *	0.27 *
O_3						1.00	0.44 *	−0.63 *
Temperature							1.00	−0.31 *
Relative humidity								1.00

* $p < 0.001$.

Table S2. Effects of PM_{10} on CHD hospitalization at different time, temperature and relative humidity *df* values.

Variables	*df*	AIC	ER	95%CI
Time	3	10379.58	2.74	(1.50~4.00)
	4	10271.01	2.19	(0.92~3.47)
	5	10194.56	1.86	(0.58~3.16)
	6	10200.45	1.95	(0.67~3.25)
	7	10173.31	1.86	(0.57~3.16)
	8*	10131.72	1.69	(0.39~3.00)
Temperature	3	10138.31	1.68	(0.39~2.99)
	4*	10131.72	1.69	(0.39~3.00)
	5	10133.41	1.71	(0.41~3.02)
	6	10134.03	1.71	(0.42~3.02)
Relative humidity	3*	10131.72	1.69	(0.39~3.00)
	4	10131.72	1.69	(0.39~3.00)
	5	10131.72	1.69	(0.39~3.00)

ER means excess risk (%); All were statistically significant, * represents the degrees of freedom used in this study.
